# Psychometric qualities of the English Coping Scales of the Stress and Coping Inventory in a representative UK sample

**DOI:** 10.1186/s40359-021-00528-3

**Published:** 2021-02-02

**Authors:** Teresa O’Rourke, Sanja Budimir, Christoph Pieh, Thomas Probst

**Affiliations:** 1 Department for Psychotherapy and Biopsychosocial Health, Danube University Krems, Krems an der Donau, Austria; 2 Department of Work, Organization and Society, Ghent University, Ghent, Belgium

**Keywords:** Coping, SCI inventory, Reliability, Validity

## Abstract

**Background:**

The Coping Scales of the Stress and Coping Inventory (SCI; Satow in Stress- und Coping-Inventar (SCI): Test- und Skalendokumentation. Stress and coping inventory. http://www.drsatow.de, 2012) are well-established German self-report scales measuring five coping styles: Positive Thinking, Active Coping, Social Support, Support in Faith, and Alcohol and Cigarette Consumption. The purpose of this study was to translate the scales into English and to psychometrically evaluate this English version of the SCI coping scales with a representative sample of the UK population.

**Methods:**

The coping scales of the SCI were forward–backward translated into English and administered to a representative sample according to age, gender, education, and region for the UK (N = 1006). Internal consistencies, factorial validity, and construct validity were assessed for both the original factor structure of the SCI, as well as a newly identified factor structure.

**Results:**

The results for the original factor structure indicated good internal consistency and construct validity. The adaptive coping styles of this version were positively correlated with resilience and negatively with perceived stress. The maladaptive coping strategy, alcohol and cigarette consumption, showed the opposite correlations. The exploratory factor analysis (EFA) of the English version resulted in a five-factor structure, but some items loaded on different factors than in the German version. These new factors were Religious Coping, Social Support, Various Coping, Alcohol and Cigarette Consumption, and Reflective Coping. The novel factors showed similar correlations to resilience and perceived stress as the original factor structure. Only religious coping did not significantly correlate to perceived stress. Confirmatory factor analysis with the original factor structure of the German SCI coping scales revealed poor model fit for the English SCI coping scales.

**Conclusion:**

The English SCI coping scales consistently and accurately measure five different coping styles. Nevertheless, the original factor structure of the SCI coping scales, when applied to an English-speaking sample, did not fit the data well. The new factor structure established by EFA is only preliminary and needs further validation in future large samples using the English version of the SCI coping scales.

## Introduction

Coping is characterized as different cognitive and behavioral patterns in dealing with external and internal demands of stressful situations [21]. According to the stress process model [35], coping strategies can modify the stress response following a stressor as well as subsequent health consequences through behavioral and physiological processes. Although coping has been well researched throughout the years (e.g. [21, 22, 38]), assessment approaches of coping strategies and behaviors vary based on the underlying definitions of coping.

Coping in general is defined as the ability to manage stressful, threatening, or burdensome demands of a situation [5]. Based on one of the most established models of coping, the transactional stress model by Lazarus and Folkman [21], coping strategies are commonly classified as either problem-oriented or emotion-oriented. Problem-oriented coping is aimed at resolving the stress-inducing situation, whereas emotion-oriented coping is aimed at reducing the emotional aftermath of a stressful situation [22]. Coping is also commonly described to be either adaptive or maladaptive, although this classification depends not only on whether an individual can successfully handle a stressful situation with a particular coping strategy or not, but also other factors such as long-term developmental consequences [38].

Adaptive coping strategies include active coping [24], social support, [39] or positive thinking [32], while maladaptive coping strategies include avoidance, social withdrawal, [16] or alcohol consumption [39]. It is important to note that alcohol consumption is not necessarily a coping strategy, as it is also seen as a leisure activity by many people [43]. Alcohol consumption can only be regarded as a coping strategy if alcohol is consumed with the explicit intention of reducing stress. Adaptive coping strategies can have protective effects on physical and mental health [24] and prevent negative health outcomes of severe and chronic stress [7, 9], such as cardiovascular disease and reduced immune function [8, 13]. Maladaptive coping strategies can have detrimental effects on physical and mental health [20, 27]. Regarding mental health, severe stress has been associated with a variety of disorders such as depression and anxiety [12, 37, 40], or somatoform disorders [44], which include somatoform autonomic dysfunctions or persistent somatoform pain disorders [46]. Furthermore, negative health beliefs about stress, i.e. believing that stress is bad for one’s health, have been associated with somatic symptoms during the experience of stress [15]. The coping strategies of social support and optimism have been shown to be inversely correlated with symptoms of stress, depression, and anxiety [39]. Religious coping can involve practices such as meditation or mindfulness, which have been associated to psychological well-being, self-esteem, and life satisfaction and can have protective effects against morbidity and mortality [41].

One of the best-known questionnaires for the assessment of coping is the “BriefCOPE” created by Carver [4], which discerns 14 different coping strategies. This structure, however, could never be confirmed by factor analyses and most factor analyses suggest three to five different coping strategies [19]. Taking these results into account, Satow [34] developed the coping scales of the Stress and Coping Inventory (SCI) in German, which are reliable and valid German scales for the measurement of stress coping styles. With 20 items, four adaptive coping styles (Positive Thinking (PT), Active Stress Coping (AS), Social Support (SS), and Support in Faith (SF)) and one maladaptive coping style (Alcohol and Cigarette Consumption (AC)) are measured. Lower scores on adaptive coping styles do not automatically equate to higher scores on maladaptive coping and vice versa. A higher score on any of the SCI coping scales represents the tendency to use this specific coping strategy in everyday life for the last month. The five-factor structure was found in the original German sample (N = 5520, 57% female). The largest age group in this sample consisted of 20 to 30 year old participants. The coping scales of the German version showed good internal consistencies in this original sample: PT (α = 0.74), AS (α = 0.74), SS (α = 0.88), SF (α = 0.78), AC (α = 0.75).

Although the SCI coping scales are a validated and reliable measure of coping, there is no English version and no validation of that in an English-speaking population yet. The purpose of the present paper was to close this gap by translating the SCI coping scales into English and assessing the psychometric properties (reliability and validity, confirmatory factor analysis) of both the original German version and the translated version with a representative sample (regarding age, gender, education, and region) of the UK population.

Based on previous research [2, 34], we hypothesized that higher scores in adaptive coping would be associated with greater resilience and lower perceived stress, whereas higher scores in maladaptive coping would be associated with higher perceived stress and less resilience. Based on the factor structure of the original German SCI coping scales, we assumed the five factors (PT, AS, SS, SF, AC) can be replicated for the English translation in the UK population.

## Methods

### Study design

The present study was based on a representative survey of the UK population. An online survey aiming at a representative sample according to age, gender, education, and region was performed with Qualtrics® [30] to measure mental health during the COVID-19 lockdown in the UK [28]. The survey started on the 21st of April 2020, 4 weeks after lockdown measures became obligatory in the UK on 24th of March 2020, and lasted 10 days.

### Sample and procedure

A representative sample according to age, gender, education, and region for the UK was recruited through Qualtrics panel, by quota sampling. Participants were registered with the Qualtrics panel and contacted by Qualtrics project team who organized and coordinated data collection. We aimed for a representative sample size according to age, gender, education, and region of at least 1000 participants. According to COSMIN risk of bias checklist [26], a “very good” sample size for factor analysis is seven times the number of items and ≥ 100. Demographic characteristics of the study sample (N = 1006) are presented in Table 1.

**Table 1:** Sample characteristics

	N (%)
*Gender*	
Women	544 (54.1)
Men	462 (45.9)
*Age*	
18–24	98 (9.7)
25–34	203 (20.2)
35–44	190 (18.9)
45–54	194 (19.3)
55–64	173 (17.2)
65 +	148 (14.7)
*Relationship status*	
Single	289 (28.7)
Living separately	18 (1.8)
Married	406 (40.4)
Divorced	75 (7.5)
In a partnership	187 (18.6)
Widowed	31 (3.1)
*Working situation*	
Not working now (and did not before lockdown)	243 (24.2)
Not working now (but did before lockdown)	235 (23.4)
Working, now in home-office	176 (17.5)
Working not in home-office	133 (13.2)
Working, now in short term work	78 (7.8)
Retired	141 (14.0)
*Income*	
< € 1.000,-	138 (13.7)
€ 1.000,- to € 2.000,-	343 (34.1)
€ 2.000,- to € 3.000,-	256 (25.4)
€ 3.000,- to € 4.000,-	147 (14.6)
> € 4.000,-	122 (12.1)
*Living situation*	
Living in a flat	202 (20.1)
Living in a flat with a balcony or terrace	56 (5.6)
Living in a house (with/without garden)	748 (74.4)

### Measures

#### Coping Scales of the Stress and Coping Inventory (SCI)

To measure the participants’ stress coping styles, we translated the coping scales (20 items) of the German SCI [34] into English. The coping scales of the SCI were translated into English using the forward–backward translation approach [1]. A bilingual researcher translated the German items into English and a different, German-speaking researcher translated these items back into German. Any discrepancies were resolved through consensus. The translated English version of the SCI coping scales is provided as an Additional file 1. A five-factor structure was found for the original German version of the SCI coping scales [34]: (1) Positive Thinking, (2) Active Stress Coping, (3) Social Support, (4) Support in Faith, and (5) Alcohol and Cigarette Consumption. The scales of the German version each consist of four items scored on a four-point Likert scale from 1 (do not agree at all) to 4 (strongly agree). For most items, higher values represent a better fit with the respective coping style. Only in one item of the scale *Alcohol and Cigarette Consumption* does a higher score indicate a worse fit with this particular coping style (“No matter how much stress I get, I would never turn to alcohol or cigarettes because of stress.”). This item has hence to be recoded before calculating the AC scale score. Higher scores on a scale level represent higher coping, although higher scores in the AC scale represent a more maladaptive coping.

#### Perceived Stress Scale (PSS)-10

To measure perceived stress within the past month, the reliable and validated 10-item perceived stress scale (PSS-10; [6]) was used. The items are scored on a Likert scale from 0 to 4, with higher scores indicating higher perceived stress. The Cronbach’s alpha was α = 0.88 in the current sample.

#### Connor-Davidson Resilience Scale (CD-RISC)-10

The reliable and valid 10-item CD-RISC [3], adapted from the original 25-item CD-RISC [10] was used to measure resilience. It consists of 10 items scored on a five-point scale from 0 to 4, with higher scores indicating greater resilience. The Cronbach’s alpha was α = 0.93 in the current sample.

### Statistical analysis

Descriptive statistics were calculated to describe the sample. Internal consistencies (Cronbach’s Alphas) were calculated to assess reliability. Construct validity was assessed by calculating Pearson correlations between the coping styles and resilience to account for convergent validity (divergent validity for the AC scale) and between the coping styles and perceived stress to account for divergent validity (convergent validity for the AC scale). An exploratory factor analysis (EFA) was conducted, as the factors in the English version were unclear. Internal consistencies, factorial validity, and construct validity were assessed for both the original factor structure of the German SCI as well as the newly identified factor structure of the English version. Descriptive statistics, construct validity, and EFA were conducted with SPSS 26. To evaluate how well the five-factor structure Satow [34] found for the German SCI coping scales could be replicated in English, a confirmatory factor analysis (CFA) with the five scales of the original German version was additionally calculated using AMOS. The model was estimated with the maximum likelihood method approach. Model fit was evaluated by using Chi-square statistic, the comparative-fit-index (CFI), and the Tucker-Lewis Index (TLI) to describe incremental fit. The root mean square error of approximation (RMSEA) was used as an absolute measure of fit. A better fit is indicated by TLI and CFI values close to 0.95 or higher and an RMSEA of 0.08 or lower [18].

## Results

### Exploratory factor analysis

An EFA was conducted on the newly developed 20 English items of the SCI coping scales with the maximum likelihood estimation method and varimax rotation. The Kaiser–Meyer–Olkin measure verified the sampling adequacy for the analysis (KMO = 0.86). Table 2 shows the factor loadings after rotation. The highest factor loading for each item is highlighted.

**Table 2:** Summary of exploratory factor analysis results for the English SCI coping scales (N = 1006)

Item	Rotated factor loading					
	1	2	3	4	5	Original scale
Factor 1 (Religious Coping)						
9. Prayer helps me deal with stress and threats	**.90**	.05	.07	.04	.13	Support in Faith
10. No matter how bad it gets, I trust in higher forces	**.85**	.10	.10	.01	.14	Support in Faith
8. Under stress and pressure, I find stability in faith	**.82**	.09	.13	.04	.23	Support in Faith
Factor 2 (Social Support)						
13. When I come under pressure, I have people who help me	.07	**.81**	.23	− .02	.08	Social Support
4. When I feel overwhelmed, there are people who build me up again	.12	**.71**	.10	− .06	.17	Social Support
15. Under stress and pressure, I find support in my partner or a good friend	.02	**.69**	.22	− .03	.10	Social Support
19. No matter how bad it gets, I have good friends that I can count on	.05	**.59**	.25	− .02	.21	Social Support
Factor 3 (various coping)						
17. Under stress and pressure, I purposefully eliminate the causes	.08	.22	**.67**	− .04	.24	Active Stress Coping
12. I do anything to prevent stress from arising in the first place	.03	.14	**.57**	− .01	.00	Active Stress Coping
16. Under stress and pressure, I simply concentrate on the positive	.14	.26	**.51**	− .18	.41	Positive Thinking
7. I try to avoid stress in advance	.04	.09	**.51**	− .05	− .00	Active Stress Coping
18. Under stress and pressure, I remember that there are greater values in life	.16	.31	**.49**	− .13	.33	Support in Faith
6. Even when I am under a lot of pressure, I do not lose my sense of humor	.08	.24	**.36**	− .15	.34	Positive Thinking
Factor 4 (Alcohol and Cigarette Consumption)						
11. When everything gets too much for me, I sometimes take to the bottle	.04	− .058	.002	**.88**	− .06	Alcohol and Cigarette consumption
14. Under stress and pressure, I relax with a glass of wine or beer in the evening	− .03	.04	.04	**.78**	.03	Alcohol and Cigarette Consumption
2. No matter how much stress I get, I would never turn to alcohol or cigarettes because of stress	.15	.12	.23	− **.59**	.18	Alcohol and Cigarette Consumption
20. If the stress gets too much, I will smoke a cigarette	.12	− .02	− .09	**.42**	.01	Alcohol and Cigarette Consumption
Factor 5 (Reflective Coping)						
5. I see stress and pressure as a positive challenge	.22	.16	.14	− .02	**.71**	Positive Thinking
1. I tell myself that stress and pressure also have their good sides	.14	.14	.05	.01	**.68**	Positive Thinking
3. I think about how I can avoid time pressure beforehand	.16	.15	.34	− .12	**.35**	Active Stress Coping
% of variance	28.17	12.37	10.38	6.95	6.13	

The items that cluster on the same factor in the English version suggest that factor 1 represents religious coping, factor 2 represents Social Support, and factor 4 represents Alcohol and Cigarette Consumption. Factors 3 (Various Coping) and 5 (Reflective Coping) are ambiguous, as items from two scales of the original German version (positive thinking and active stress coping) load on both of these factors. Moreover, there are double and triple loadings for the items loading on these new factors and they load under their initial factors as found in the German version as well. Item 18 “Under stress and pressure, I remember that there are greater values in life”, which is included in the scale support in faith in the original German version, furthermore shows the highest factor loading on factor 3. As items from three scales of the original German version load on factor 3, we refer to this new factor as Various Coping. The items that load on factor 5 all address thinking about stress. We therefore named this factor Reflective Coping.

### Confirmatory factor analysis

**Fig. 1:**
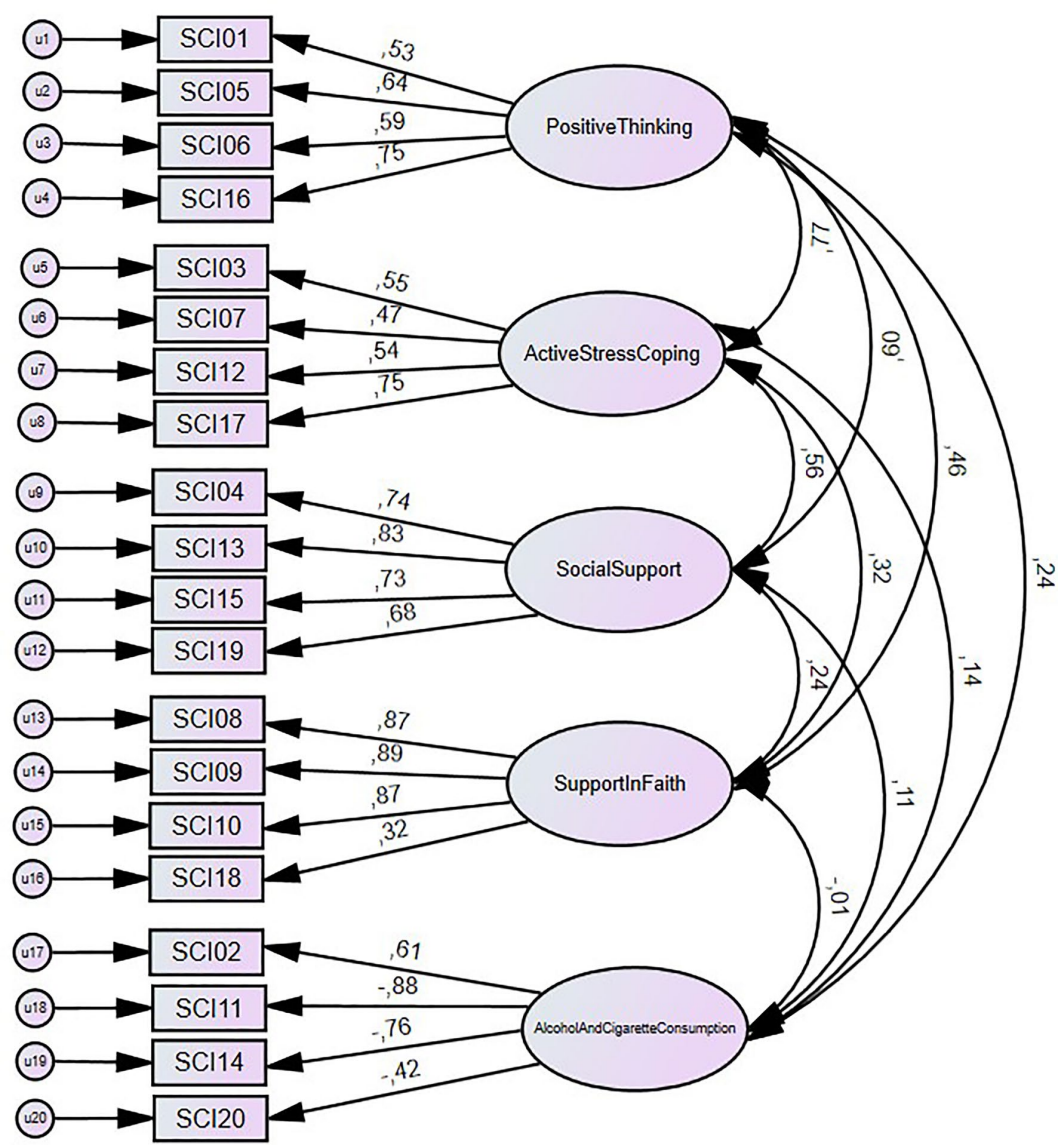
Standardized regression weights of the five-factor model evaluated in the confirmatory factor analysis

The five-factor structure of the SCI coping scales as found for the original German version by Satow [34] was evaluated via CFA for the English version. The model and corresponding standardized regression weights are depicted in Fig. 1. Both incremental fit indices (CFI = 0.86, TLI = 0.83) and absolute measures of fit indices (RMSEA = 0.086) revealed poor model fit (χ^2^(159, N = 1006) = 1333.32, *p* < 0.001) of the original five-factor model of the German SCI coping scales [34]. Standardized factor loadings of Positive Thinking ranged from 0.44 to 0.77,those of Active Stress Coping from 0.54 to 0.76; those of Social Support from 0.67 to 0.83, those of Support in Faith from 0.32 to 0.83, and the standardized factor loadings of Alcohol and Cigarette Consumption ranged from − 0.42 to -0.89.

#### Options to increase model fit

**Fig. 2:**
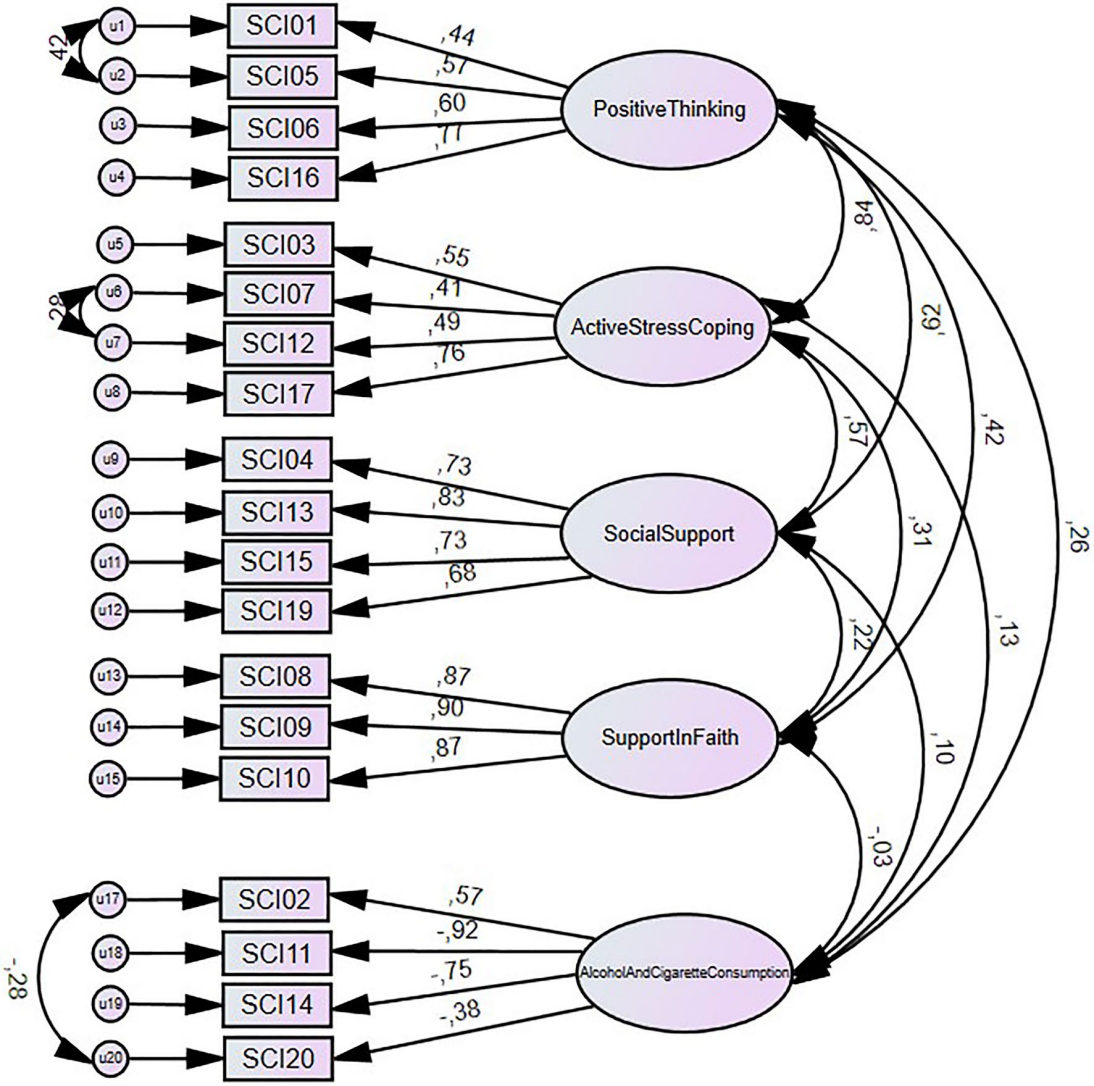
Standardized regression weights of the five-factor model after improving model fit

The model fit increased significantly when item 18 was deleted and the error terms of items 1 and 5, 7 and 12, as well as 2 and 20 were covaried, respectively. Both incremental fit indices (CFI = 0.94, TLI = 0.92) and absolute measures of fit indices (RMSEA = 0.06; χ^2^(139, N = 1006) = 636.01, *p* < 0.001) showed good model fit after this procedure. The improved model is depicted in Fig. 2.

### Reliability

When looking at the factor structure taken from the *original German version*, the scales of the SCI showed the following internal consistencies (Cronbach’s Alphas) in this sample: *PT* (α = 0.73), *AS* (α = 0.68), *SS* (α = 0.83), *SF* (α = 0.83) and *AC* (α = 0.76). These are similar to the Cronbach’s alphas of the German SCI coping scales [34]: *PT* (α = 0.74), *AS* (α = 0.74), *SS* (α = 0.88), *SF* (α = 0.78), *AC* (α = 0.75). The reliability analysis also revealed that deleting item 18 would increase Cronbach’s Alpha of the scale *Support in Faith* to α = 0.91.

When looking at the new factor structure obtained from the EFA *for the English version*, the coping scales showed the following internal consistencies (Cronbach’s Alphas): factor 1/Religious Coping (α = 0.91), factor 2/Social Support (α = 0.83), factor 3/Various Coping (α = 0.78), factor 4/Alcohol and Cigarette Consumption (α = 0.76), factor 5/Reflective Coping (α = 0.69).

### Construct validity

Table 3 depicts the means and standard deviations of the five-factor structure of the original German version as reported by Satow [34] as well as the correlations between these five SCI coping scales, resilience, and perceived stress. Positive thinking, active stress coping, support in faith, and social support showed a significant positive correlation with resilience, with the highest correlation between positive thinking and resilience and significant negative correlations with perceived stress. The coping style alcohol and cigarette consumption showed significant small negative correlations with the other coping styles and resilience and a significant moderate positive correlation with perceived stress.

**Table 3:** Means, Standard Deviations, and Correlations between coping scales with original factor structure and mental health scales (N = 1006)

	M	SD	1	2	3	4	5	6	7
1. Positive Thinking	10.47	2.42	–						
2. Alcohol and Cigarette Consumption	7.79	3.18	− .19**	–					
3. Active Stress Coping	11.26	2.05	.50**	− .17**	–				
4. Support in Faith	9.11	3.09	.47**	− 0.05	.33**	–			
5. Social Support	11.02	2.49	.66**	− .11**	.45**	.37**	–		
6. CD-RISC	24.56	8.12	.67**	− .21**	.45**	.31**	.53**	–	
7. PSS-10	17.71	7.94	− .50**	.31**	− .33**	− .14**	− .41**	− .61**	–

**p < 0.001, CD-RISC: Connor-David Resilience Scale 10; PSS-10: Perceived Stress Scale 10

Table 4 shows the means and standard deviations of the five-factor factor structure obtained from the above mentioned EFA for the translated English version and the correlations between these five coping scales, resilience, and perceived stress. All adaptive coping scales showed significant positive correlations with resilience, with the highest correlation between factor 3 and resilience. The adaptive coping scales furthermore all showed significant negative correlations with perceived stress, except for the correlation between factor 1 (Religious Coping) and perceived stress, which did not reach statistical significance. The maladaptive coping scale Alcohol and Cigarette Consumption, which consists of the same items in the new factor structure found for the English translation as in the original German version, showed significant small negative correlations with the other coping scales, except for Religious Coping. Furthermore, it showed a small negative correlation with resilience and a moderate positive correlation with perceived stress.

**Table 4:** Means, Standard Deviations, and Correlations between coping scales with new factor structure and mental health scales (N = 1006)

	M	SD	1	2	3	4	5	6	7
1. Factor 1 (Religious Coping)	6.18	2.79	–						
2. Factor 2 (Social Support)	11.48	2.68	.20**	–					
3. Factor 3 (Various Coping)	17.13	3.10	.28**	.52**	–				
4. Factor 4 (Alcohol and Cigarette Consumption)	7.79	3.18	− .003	− .11**	− .22**	–			
5. Factor 5 (Reflective Coping)	7.54	1.87	.38**	.37**	.51**	− .13**	–		
6. CD-RISC	24.56	8.12	.21**	.43**	.63**	− .21**	.53**	–	
7. PSS-10	17.71	7.94	− .06	− .32**	− .46**	.31**	− .38**	− .61**	–

**p < 0.001, CD-RISC: Connor-David Resilience Scale 10; PSS-10: Perceived Stress Scale 10

## Discussion

This study translated the coping scales of the German SCI into English and examined the psychometric qualities of the English SCI coping scales in a representative UK sample, to find out whether the translated version provides a useful tool for measuring coping styles in English-speaking populations. First EFA was used to explore the factor-structure of the English SCI coping scales and then CFA was applied to evaluate how well the original five-factor structure for the German version as reported by Satow [34] can be replicated for the English version. Similar to the German version, the EFA performed with the English version found a five-factor structure, although some items loaded on different factors, which resulted in two ambiguous factors, both containing items from the original factors positive thinking and active stress coping. CFA revealed poor model fit for the original five factor structure that has been reported for the German SCI scales by Satow [34]. The results of the current study indicated good internal consistency for both the original (German version) as well as the newly found (English version) factor structure, with Cronbach’s alphas similar to those of the German SCI [34]. Whereas the original coping scales consist of four items each, the new coping scales do not contain the same number of items anymore. For example, factor 1 (Religious Coping) consists of only three items, while factor 3 (Various Coping) now contains six items. Cronbach’s Alphas tend to increase with the number of items in a scale, which has to be considered when interpreting these results. Nevertheless, while the three-item factor 5 (Reflective Coping) has a rather low α of 0.69, the three-item factor 1 has an α of 0.91, which is even higher than that of the respective original German scale with four items. Construct validity of the new factor structure obtained from EFA for the English version is supported by significant correlations showing convergent and divergent validity. Coping styles considered as adaptive correlated positively with resilience and negatively with perceived stress, except for one factor. As Factor 1 (Religious Coping) did not significantly correlate with perceived stress, the hypothesis regarding divergent validity could not be confirmed for this factor. The coping style alcohol and cigarette consumption, which is considered as maladaptive, correlated positively with perceived stress and negatively with resilience. The original factor structure reported by Satow [34] showed similar correlations in our English-speaking sample. Coping scales considered as adaptive correlated positive with resilience and negative correlations with perceived stress. Alcohol and Cigarette Consumption again correlated negatively with the other coping scales and resilience and positively with perceived stress. These results for convergent/divergent validity correspond to the correlations reported in the original German sample by Satow [34]: the SCI scales Positive Thinking, Social Support, and Active Stress Coping correlated negatively with stress symptoms, and Alcohol and Cigarette consumption correlated positively with stress symptoms in the original German sample [34].

Similar to our results, prior research shows that adaptive coping strategies are characterized by better coping results, such as better psychological adjustment [33] or well-being [41], while maladaptive coping strategies can increase perceived stress [16]. Furthermore, a lack of adaptive coping strategies has been associated with chronic stress [31]. A variety of studies show that alcohol consumption can be predicted by high stress levels (e.g. [11, 45]). In contrast to our results, the consumption of alcohol as a mean to cope with stressful situations can also have stress-reducing effects, although these typically are only short-term effects [23]. A negative relationship between alcohol consumption and stress is supported by research in the long-term [25]. Chronic alcohol abuse negatively affects several neurological and physiological functions, including the hypothalamic–pituitary–adrenal (HPA)-axis, which can lead to long-term stress dysregulation [17]. The positive effects of alcohol consumption furthermore seem to depend to some extent on personality traits such as extraversion [14].

Regarding coping and resilience, our results replicate previous findings. Campbell-Sills et al. [2] found that task-oriented coping strategies, intended to represent adaptive coping, were positively related to resilience, while emotion-oriented coping strategies, intended to represent maladaptive coping, were associated with lower resilience. A study on coping and resilience in competitive sport also reported positive correlations between task-oriented coping strategies and resilience, while disengagement- and distraction-oriented coping strategies correlated negatively with resilience [36]. Syed et al. [42] on the other hand, discuss how task orientation in the workforce can also negatively impact psychosocial factors and even lead to increased stress. Smith et al. [39] found resilience to be positively correlated to social support and active coping and negatively correlated to substance use. Furthermore, resilience was negatively correlated with perceived stress.

Although the results indicate good reliability and construct validity, the factorial validity was problematic. Similar to the original scale structure found for the German version [34], the EFA performed with the English version resulted in a five-factor structure. However, the resulting factor loadings differed to some extent to those from the original study with the German version and some items loaded on different factors than in the original German version found by Satow [34]. In line with the conceptualization of adaptive and maladaptive coping strategies, the first and second factors of the English version, obtained from EFA, represent the adaptive coping styles support in faith and social support and the fourth factor represents the maladaptive coping style alcohol and cigarette consumption. The third and fifth factors seem to represent adaptive coping styles as well. However, they are more difficult to interpret, as they both hold items from the scales positive thinking and active stress coping. The third factor was named Various Coping as it contained items from three different scales which did not seem to have an obvious common topic. The fifth factor was named Reflective Coping as all items seemed to address some form of reflection about stress.

Furthermore, the originally found five-factor structure in the German version showed poor model fit when tested in CFA with the English items. The model fit could be improved by covarying some of the error terms and deleting item 18. Both the reliability analysis and the EFA showed this item to be problematic as well. It is relevant to note that this item had the lowest discriminatory value (0.43) as well as the lowest factor loading (0.35) of all the items in the original study as well [34].

One reason for the divergence of these results for the English version to the original findings for the German version could be that the coping behavior of our sample was influenced by the COVID-19 lockdown measures. It is possible that opportunities for active coping strategies were limited by lockdown restrictions and some individuals resorted to different forms of coping, such as positive thinking. Moreover, it could be that some forms of intentional positive thinking were seen as a form of active coping. Similarly, it is possible that some forms of religious coping, such as collective prayer in a house of worship, were restricted by lockdown measures. Furthermore, the problematic item 18 “Under stress and pressure, I remember that there are greater values in life” does not explicitly refer to faith, prayer, or higher forces like the other three items of the support in faith factor found in the German version [34], that clustered together on factor 1 (Religious Coping) in the factor structure found via EFA for the English version. It is possible that the participants in the British sample did not associate greater values to religious coping, in contrast to the German-speaking original sample. Interpreted in a non-religious way, this item can also be assigned to positive thinking. This inconsistency might be resolved by exploring the participants’ specific interpretations of this item. It is important to note that these deliberations are highly speculative, however, and should be explored further in qualitative studies.

### Limitations

Various limitations should be considered when interpreting these results. The cross-sectional design and calculated correlations do not allow for causal conclusions. To investigate causal relationships between coping styles and stress as well as resilience, longitudinal studies are needed.

Furthermore, as the participants in this study were exposed to a particularly unusual situation—COVID-19 lockdown measures and restrictions—it is possible that the results are different than they would have been under normal circumstances, since coping behavior might differ during lockdown. During the COVID-19 lockdown measures, which became obligatory on the 24^th^ of March 2020 in the UK, leaving the house was only allowed in the following exceptions: shopping for food and other necessities, exercising alone or with someone from the same household, leaving the house for medical reasons, including providing care to others, and commuting to and from work. Studies on mental health during lockdown in the UK revealed that psychological distress increased during lockdown in comparison to pre-COVID times [28, 29]. The factor structure of the English SCI coping scales might therefore differ from the German SCI coping scales, which were analyzed under ordinary circumstances. It is furthermore possible, that the factor structure could have looked different if the data had been gathered in an ordinary situation, outside of the COVID-19 pandemic. However, it is unclear how this particular situation affected the results of the present study. The new factor structure found in EFA for the English version is only preliminary and needs to be evaluated with CFA in future studies with large new samples.

## Conclusion

In conclusion, the English SCI coping scales are reliable for assessing coping styles. Although the construct validity is given, the original five-factor structure from the German version showed poor model fit when tested in CFA with the English items. The new factor structure found via EFA for the English version needs to be evaluated in the future with CFA in new samples. It is also important to consider that the participants in this study were exposed to an extreme situation, which might affect comparability of results.

## Supplementary Information

**Additional file 1.** Translated English Version of the SCI coping scales.

## Data Availability

All relevant data are provided upon reasonable request (Teresa.orourke@donau-uni.ac.at).

## Abbreviations

AC: Alcohol and Cigarette Consumption
AS: Active Stress Coping
CD-RISC: Connor-Davidson Resilience Scale
CFA: Confirmatory factor analysis
CFI: Comparative-Fit-Index
COVID: Coronavirus Disease
EFA: Exploratory factor analysis
HPA: Hypothalamic–pituitary–adrenal (Axis)
KMO: Kaiser–Meyer–Olkin measure
PSS: Perceived Stress Scale
PT: Positive Thinking
RMSEA: Root mean square error of approximation
SCI: Stress and Coping Inventory
SF: Support in Faith
SS: Social Support
TLI: Tucker–Lewis Index
