# Supplementary material for: Psychometric qualities of the English Coping Scales of the Stress and Coping Inventory in a representative UK sample
Source: BMC Psychol. 2021 Feb 2;9:23. doi: 10.1186/s40359-021-00528-3 (PMC7851809; doi:10.1186/s40359-021-00528-3)
Supplement: Supplementary file 1 — Additional file 1. Translated English Version of the SCI coping scales. [file 40359_2021_528_MOESM1_ESM.docx]

Translated English version of the SCI coping scales

|  | strongly disagree | disagree | agree | strongly agree |
| --- | --- | --- | --- | --- |
| 1. I tell myself that stress and pressure also have their good sides. | ° | ° | ° | ° |
| 2. No matter how much stress I get, I would never turn to alcohol or cigarettes because of stress. | ° | ° | ° | ° |
| 3. I think about how I can avoid time pressure beforehand. | ° | ° | ° | ° |
| 4. When I feel overwhelmed, there are people who build me up again. | ° | ° | ° | ° |
| 5. I see stress and pressure as a positive challenge. | ° | ° | ° | ° |
| 6. Even when I am under a lot of pressure, I do not lose my sense of humor. | ° | ° | ° | ° |
| 7. I try to avoid stress in advance. | ° | ° | ° | ° |
| 8. Under stress and pressure, I find stability in faith. | ° | ° | ° | ° |
| 9. Prayer helps me deal with stress and threats. | ° | ° | ° | ° |
| 10. No matter how bad it gets, I trust in higher forces. | ° | ° | ° | ° |
| 11. When everything gets too much for me, I sometimes take to the bottle. | ° | ° | ° | ° |
| 12. I do anything to prevent stress from arising in the first place. | ° | ° | ° | ° |
| 13. When I come under pressure, I have people who help me. | ° | ° | ° | ° |
| 14. Under stress and pressure, I relax with a glass of wine or beer in the evening. | ° | ° | ° | ° |
| 15. Under stress and pressure, I find support in my partner or a good friend. | ° | ° | ° | ° |
| 16. Under stress and pressure, I simply concentrate on the positive. | ° | ° | ° | ° |
| 17. Under stress and pressure, I purposefully eliminate the causes. | ° | ° | ° | ° |
| 18. Under stress and pressure, I remember that there are greater values in life. | ° | ° | ° | ° |
| 19. No matter how bad it gets, I have good friends that I can count on. | ° | ° | ° | ° |
| 20. If the stress gets too much, I will smoke a cigarette. | ° | ° | ° | ° |
